# Supplementary material for: Origin, genetic diversity and evolution of Andaman local duck, a native duck germplasm of an insular region of India
Source: PLoS One. 2021 Feb 9;16(2):e0245138. doi: 10.1371/journal.pone.0245138 (PMC7872295; doi:10.1371/journal.pone.0245138)
Supplement: S1 Table — (DOCX) [file pone.0245138.s001.docx]

Table S1: Details of sampling location of Andaman Local Duck

| S. No. | Name of the Village | Latitude | Longitude | Samples (n) |
| --- | --- | --- | --- | --- |
| North Andaman | | | | |
| 1 | Shyam Nagar | 13°23'36.44"N | 92°55'53.16"E | 3 |
| 2 | Madhupur | 13°15'49.06"N | 92°57'54.21"E | 3 |
| 3 | Durgapur | 13°16'21.15"N | 93°1'59.94"E | 3 |
| 4 | Shibpur | 13°14'28.37"N | 93°2'2.65"E | 3 |
| 5 | Khudirampur | 13°14'9.79"N | 92°58'1.32"E | 3 |
| 6 | Nischintapur | 13°12'46.54"N | 92°52'17.45"E | 3 |
| 7 | Kishorinagar | 13°9'18.33"N | 92°52'48.57"E | 3 |
| 8 | Nabagram | 13°9'46.91"N | 92°56'33.25"E | 3 |
| 9 | Kalighat | 13°6'49.94"N | 92°57'36.21"E | 3 |
| Middle Andaman | | | | |
| 10 | Webi | 12°54'43.26"N | 92°53'54.53"E | 3 |
| 11 | Lucknow | 12°51'39.22"N | 92°52'57.93"E | 3 |
| 12 | Pahalgoan | 12°49'53.88"N | 92°52'24.84"E | 3 |
| 13 | Karmatang | 12°50'50.10"N | 92°56'11.55"E | 3 |
| 14 | Chainpur | 12°44'33.39"N | 92°49'5.98"E | 3 |
| 15 | Billiground | 12°40'9.32"N | 92°52'57.55"E | 3 |
| 16 | Shantipur | 12°38'27.25"N | 92°54'6.61"E | 3 |
| 17 | Kaushalyanagar | 12°32'28.12"N | 92°49'29.41"E | 3 |
| 18 | Parnasala | 12°31'1.63"N | 92°54'40.46"E | 3 |
| 19 | Bakultala | 12°30'14.39"N | 92°51'44.91"E | 3 |
| 20 | Nimbutala | 12°30'0.79"N | 92°57'42.76"E | 3 |
| 21 | Desharatpur | 12°29'38.58"N | 92°55'12.60"E | 3 |
| 22 | Long Island | 12°23'47.36"N | 92°56'6.27"E | 3 |
| 23 | Kadamtala | 12°20'43.11"N | 92°46'22.89"E | 3 |
| 24 | Nilambur | 12°10'32.72"N | 92°47'15.33"E | 2 |
