# Supplementary material for: Origin, genetic diversity and evolution of Andaman local duck, a native duck germplasm of an insular region of India
Source: PLoS One. 2021 Feb 9;16(2):e0245138. doi: 10.1371/journal.pone.0245138 (PMC7872295; doi:10.1371/journal.pone.0245138)
Supplement: S2 Table — (DOCX) [file pone.0245138.s002.docx]

Table S1: Details of the sequences used in the present study

| **ACC No** | **Country** | **Reference** |
| --- | --- | --- |
| NC_009684 | - | Reference sequence |
| MK854486- MK854556 | India | Our sequence |
| KJ823277 | Italy | GenBank |
| KJ823276 | Italy | GenBank |
| KJ823275 | Italy | GenBank |
| KJ823274 | Italy | GenBank |
| KJ823260 | Russia | GenBank |
| KJ823259 | Russia | GenBank |
| JN030157 | Russia | GenBank |
| JN030156 | Russia | GenBank |
| JN030155 | Russia | GenBank |
| JN030154 | Russia | GenBank |
| MK425381 | Canada | GenBank |
| MK425378 | Canada | GenBank |
| MK425377 | Canada | GenBank |
| MK425376 | Canada | GenBank |
| MK425375 | Canada | GenBank |
| JN030014 | Canada | GenBank |
| JN030013 | Canada | GenBank |
| EF126732 | China | GenBank |
| EF126731 | China | GenBank |
| EF126730 | China | GenBank |
| EF126729 | China | GenBank |
| EF126728 | China | GenBank |
| EF126715 | China | GenBank |
| EF126708 | China | GenBank |
| EF126707 | China | GenBank |
| EF126702 | China | GenBank |
| KJ823273 | Finland | GenBank |
| KJ823272 | Finland | GenBank |
| KJ823271 | Finland | GenBank |
| KJ823261 | Finland | GenBank |
| GQ922102 | UP, India | GenBank |
| GQ922101 | UP, India | GenBank |
| GQ922100 | UP, India | GenBank |
| GQ922099 | UP, India | GenBank |
| GQ922098 | UP, India | GenBank |
| GQ922084 | Kerala, India | GenBank |
| GQ922083 | Kerala, India | GenBank |
| GQ922082 | Kerala, India | GenBank |
| GQ922081 | Kerala, India | GenBank |
| GQ922080 | Kerala, India | GenBank |
| GQ922079 | Kerala, India | GenBank |
| GQ922078 | Kerala, India | GenBank |
| GQ922077 | Kerala, India | GenBank |
| KX756175 | Indonesia | GenBank |
| KX756174 | Indonesia | GenBank |
| KX756172 | Indonesia | GenBank |
| KX756171 | Indonesia | GenBank |
| KX756170 | Indonesia | GenBank |
| KX756169 | Indonesia | GenBank |
| KX756168 | Indonesia | GenBank |
| KX756167 | Indonesia | GenBank |
| KX756166 | Indonesia | GenBank |
| KX756165 | Indonesia | GenBank |
| KX756164 | Indonesia | GenBank |
| KX756163 | Indonesia | GenBank |
| KX756162 | Indonesia | GenBank |
| KX756161 | Indonesia | GenBank |
| KX756158 | Indonesia | GenBank |
| KX712255 | Indonesia | GenBank |
| KX712254 | Indonesia | GenBank |
| KX712253 | Indonesia | GenBank |
| KX756176 | Indonesia | GenBank |
| KX712252 | Indonesia | GenBank |
| KU845299 | South Korea | GenBank |
| KU845298 | South Korea | GenBank |
| KU845297 | South Korea | GenBank |
| KU845296 | South Korea | GenBank |
| KU845295 | South Korea | GenBank |
| KU845294 | South Korea | GenBank |
| EU013957 | Thailand | GenBank |
| EU013955 | Thailand | GenBank |
| EU013954 | Thailand | GenBank |
| EU013953 | Thailand | GenBank |
| EU013952 | Thailand | GenBank |
| EU013951 | Thailand | GenBank |
| EU013950 | Thailand | GenBank |
| EU013949 | Thailand | GenBank |
| EU013948 | Thailand | GenBank |
| EU013956 | Thailand | GenBank |
| AY928900 | USA | GenBank |
| AY928899 | USA | GenBank |
| MN734522 | USA | GenBank |
| MN734521 | USA | GenBank |
| MN734520 | USA | GenBank |
| MN734510 | USA | GenBank |
| MF069248 | China | GenBank |
| KJ833587 | China | GenBank |
| KJ739616 | China | GenBank |
| KJ689447 | China | GenBank |
| HM010684 | China | GenBank |
| FJ167857 | China | GenBank |
| EU755252 | China | GenBank |
| KX592536 | China | GenBank |
| KJ794187 | China | GenBank |
| KJ778676 | China | GenBank |
| KJ833586 | China | GenBank |
| JN030151 | Norway | GenBank |
| JN030150 | Norway | GenBank |
| JN030149 | Norway | GenBank |
| JN030148 | Norway | GenBank |
| JN030129 | The Netherlands | GenBank |
| JN030128 | The Netherlands | GenBank |
| JN030127 | The Netherlands | GenBank |
| JN030126 | The Netherlands | GenBank |
| JN030106 | Greenland | GenBank |
| JN030105 | Greenland | GenBank |
| JN030104 | Greenland | GenBank |
| JN030103 | Greenland | GenBank |
| JN030084 | Great Britain | GenBank |
| JN030083 | Great Britain | GenBank |
| JN030082 | Great Britain | GenBank |
| JN030081 | Great Britain | GenBank |
| JN030063 | Estonia | GenBank |
| JN030062 | Estonia | GenBank |
| JN030061 | Estonia | GenBank |
| JN030060 | Estonia | GenBank |
| JN030041 | Germany | GenBank |
| JN030040 | Germany | GenBank |
| JN030039 | Germany | GenBank |
| JN030038 | Germany | GenBank |
| JN029980 | Austria | GenBank |
| JN029979 | Austria | GenBank |
| JN029978 | Austria | GenBank |
| JN029977 | Austria | GenBank |
